# Supplementary figures and images for: Perturbed fatty-acid metabolism is linked to localized chromatin hyperacetylation, increased stress-response gene expression and resistance to oxidative stress
Source: PLoS Genet. 2023 Jan 10;19(1):e1010582. doi: 10.1371/journal.pgen.1010582 (PMC9870116; doi:10.1371/journal.pgen.1010582)

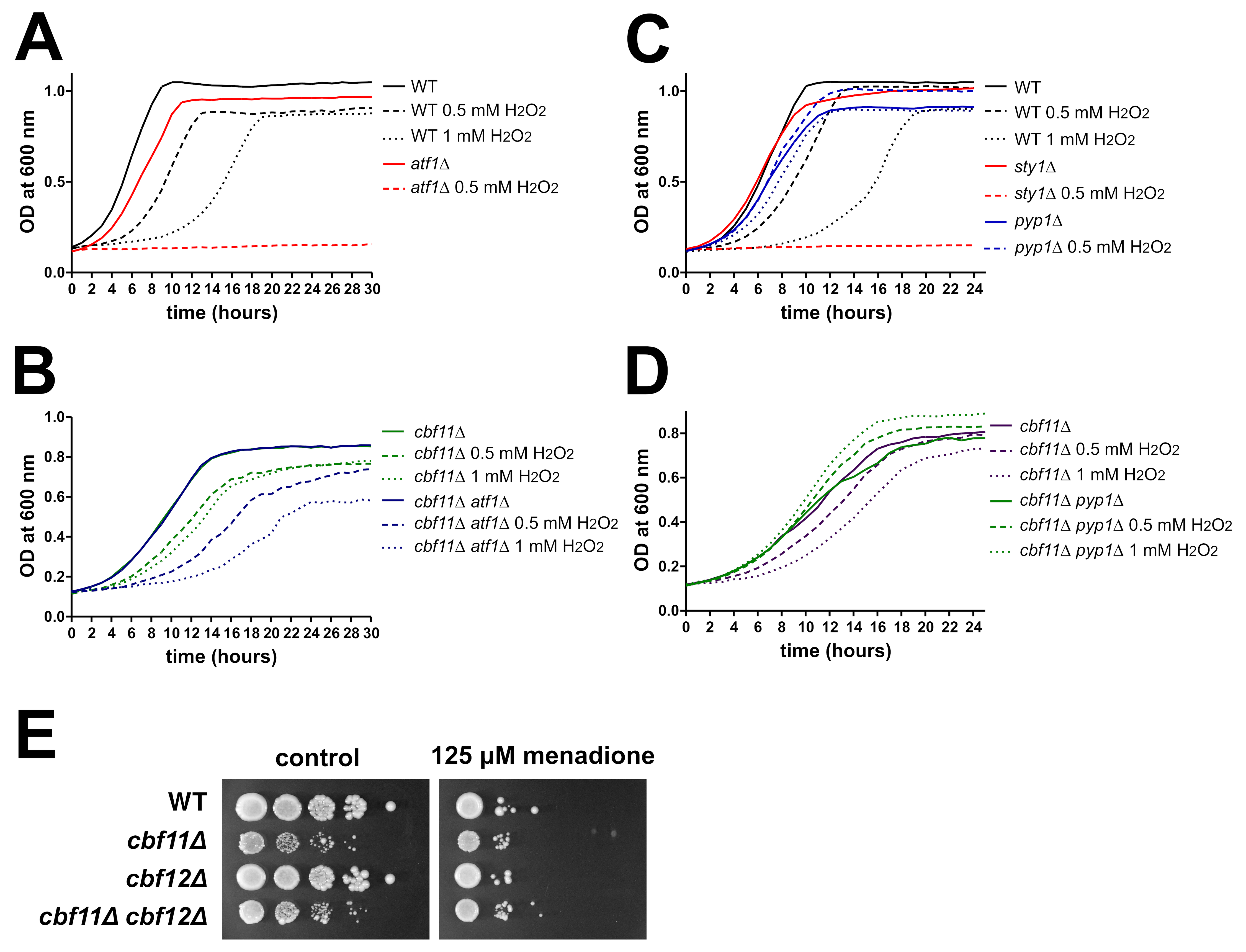

Supplement: S1 Fig — (A-D) Growth curves of stress-related mutants in the presence or absence of the indicated concentrations of H2O2 in YES medium. The pyp1Δ and sty1Δ strains represent strongly resistant and strongly sensitive controls, respectively. (E) Survival and growth under superoxide stress of WT, cbf11Δ, cbf12Δ and cbf11Δ cbf12Δ cultures spotted on YES plates containing 125 μM menadione. (TIF) [file pgen.1010582.s001.tif]

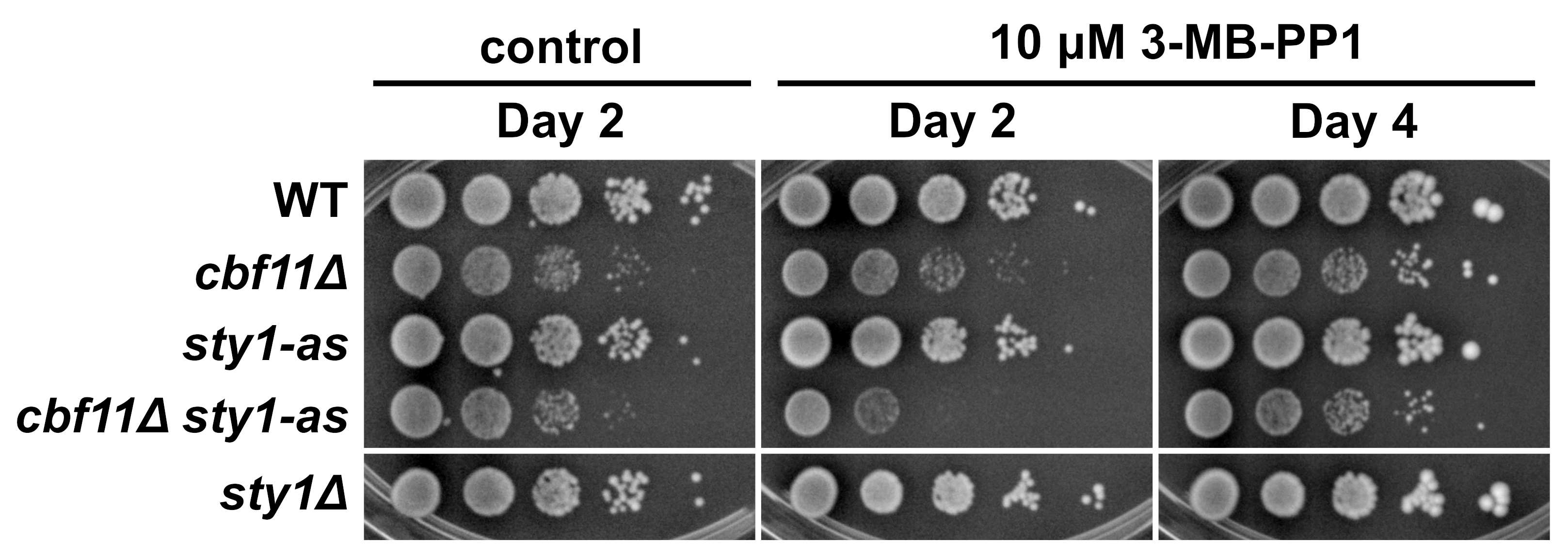

Supplement: S2 Fig — Exponentially growing WT, cbf11Δ, sty1-as, cbf11Δ sty1-as, and sty1Δ cultures were spotted on YES plates containing 10 μM Sty1-as inhibitor 3-MB-PP1 and incubated for the indicated number of days. (TIF) [file pgen.1010582.s002.tif]

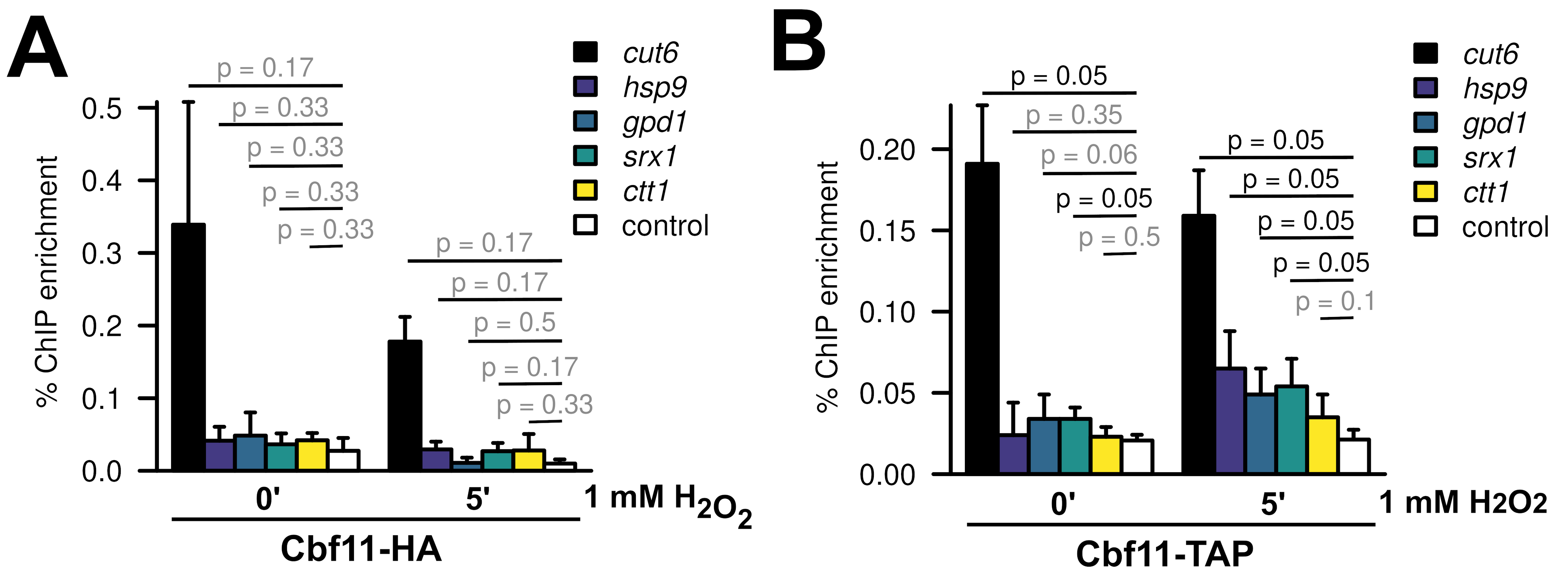

Supplement: S3 Fig — (A,B) Recruitment of Cbf11-HA and Cbf11-TAP, respectively, to known Atf1 binding sites in the indicated stress gene promoters was analyzed by ChIP-qPCR in cells treated or not with 1 mM H2O2 for 5 min in EMM medium. The cut6 promoter is a positive control for Cbf11 binding [15]; “control” is a locus with no expected Cbf11 binding. Mean and SD values of two (A) and three (B) independent replicates are shown. One-sided Mann-Whitney U test was used to determine statistical significance. (TIF) [file pgen.1010582.s003.tif]

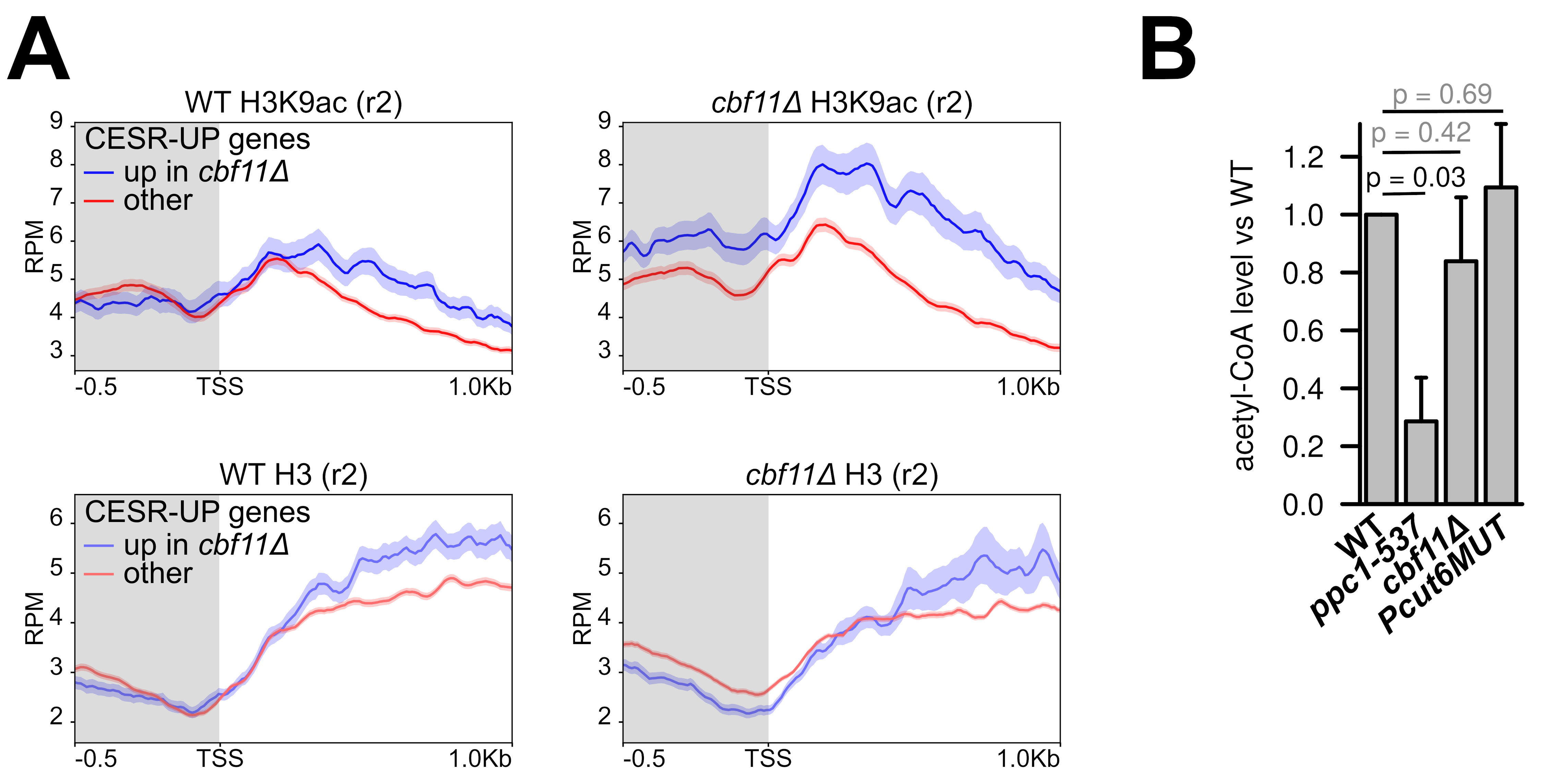

Supplement: S4 Fig — (A) Average gene profiles of total H3 (bottom panels) and acetylated H3K9 (top panels) occupancy at stress-gene regions in WT and cbf11Δ cells, respectively. Genes upregulated as part of the core environmental stress response (CESR-UP, [9]) have been divided into those showing upregulation in untreated cbf11Δ cells (blue; n = 94, [15]) and the rest (red; n = 441). Average profile of all fission yeast genes is also shown for comparison (black; n = 6952). The curves represent mean RPM (reads per million mapped reads) values ± SEM. The promoter region is shaded. TSS—transcription start site. (B) Total cellular acetyl-CoA levels in WT, ppc1-537, cbf11Δ and Pcut6MUT cell extracts were determined by LC-MS. Mean and SD values of four independent replicates for ppc1-537 and five independent replicates for all other strains tested are shown. Two-sided Mann-Whitney U test was used to determine statistical significance. (TIF) [file pgen.1010582.s004.tif]

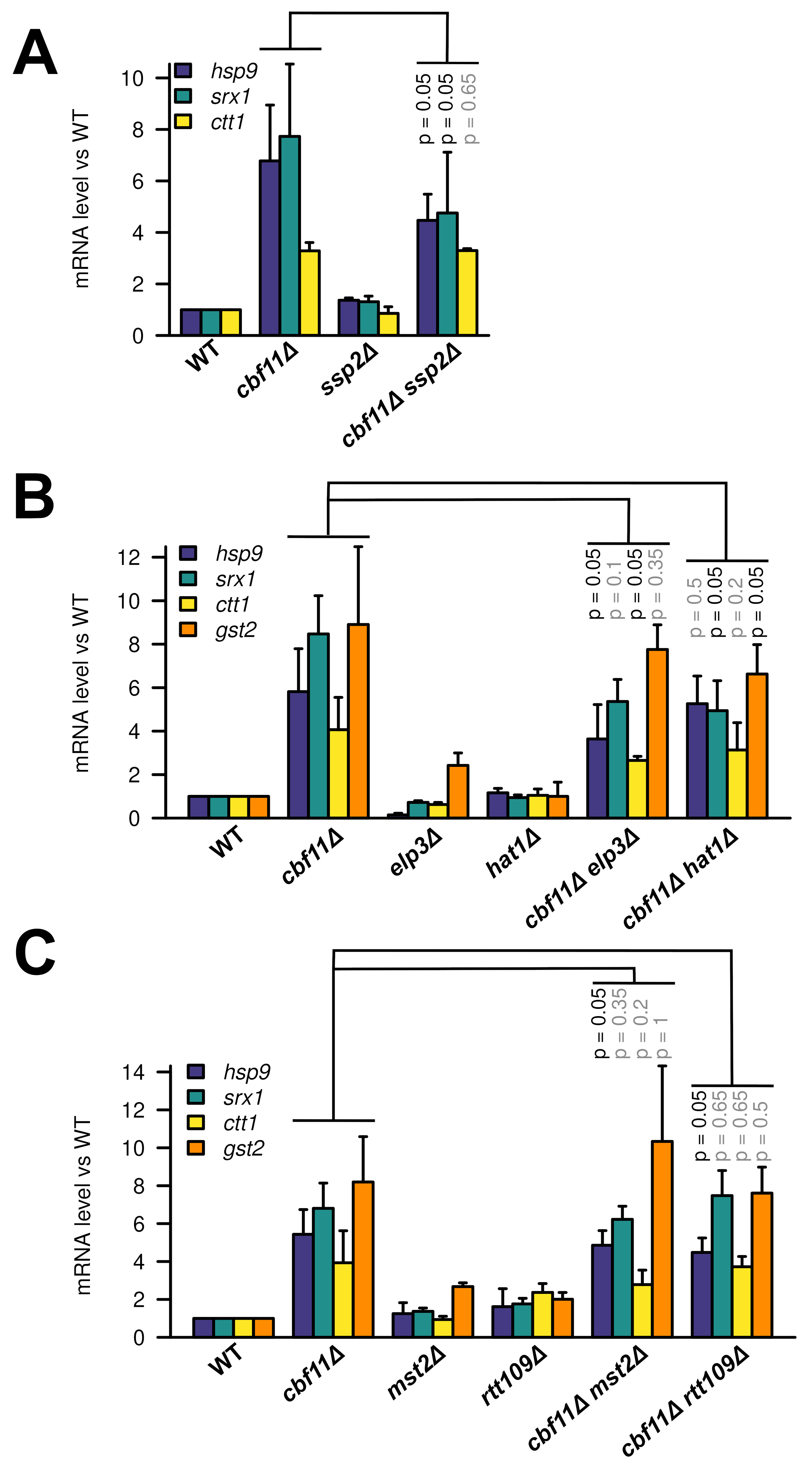

Supplement: S5 Fig — (A,B,C) Expression of the indicated stress genes in cells growing in YES medium was analyzed by RT-qPCR. Mean and SD values of three independent replicates are shown. One-sided Mann-Whitney U test was used to determine statistical significance. (TIF) [file pgen.1010582.s005.tif]
