# Supplementary material for: Perturbed fatty-acid metabolism is linked to localized chromatin hyperacetylation, increased stress-response gene expression and resistance to oxidative stress
Source: PLoS Genet. 2023 Jan 10;19(1):e1010582. doi: 10.1371/journal.pgen.1010582 (PMC9870116; doi:10.1371/journal.pgen.1010582)
Supplement: S1 Text — Table A–List of oligonucleotides; Table B–List of plasmids; Table C–List of strains. (DOCX) [file pgen.1010582.s006.docx]

**S1 Text**

**Table A – List of oligonucleotides**

| **ID** | **Sequence** | **Experiment** |
| --- | --- | --- |
| MP137 | TCCTCATGCTATCATGCGTCTT | RT-qPCR *act1* (fwd) |
| MP138 | CCACGCTCCATGAGAATCTTC | RT-qPCR *act1* (rev) |
| MP169 | GGTCTATGTTCCCACTGTTT | RT-qPCR *rho1* (fwd) |
| MP170 | CTTCTTGTCCAGCCGTA | RT-qPCR *rho1* (rev) |
| MaP173 | CCGACCCTTCATTAACATTAC | RT-qPCR *atf1* (fwd) |
| MaP174 | CCGTTGGCATATCAGAATTAG | RT-qPCR *atf1* (rev) |
| PD01 | CCGACGACATTACCAAGTA | RT-qPCR *ctt1* (fwd) |
| PD02 | ACGAGCAGTATCAGGAGTA | RT-qPCR *ctt1* (rev) |
| PD05 | TGCCTTCTTATCCCTCCT | RT-qPCR *pyp2* (fwd) |
| PD06 | ACGACGTTGCTGGATTTA | RT-qPCR *pyp2* (rev) |
| AJ33 | aaaatctagaGGGAACGTGTCAAAGATGCC | *mga2* KO cloning primer, upstream/outside, fwd (XbaI) |
| AJ34 | aaaactcgagTTGAGAGTACTTTGAAATGCAATAGA | *mga2* KO cloning primer, upstream/inside, rev (XhoI), product size 738 bp (with AJ33) |
| AJ35 | aaaatctagaGCAGATCGCATACCGGTTCA | *mga2* KO cloning primer, downstream/outside, rev (XbaI) |
| AJ36 | aaaaagatctTTTGACGAACAACCATGC | *mga2* KO cloning primer, downstream/inside, fwd (BglII), product size 424 bp (with AJ35) |
| AJ37 | TCTGTTGGCCGGAATTTGG | *mga2* KO checking primer, upstream, fwd, product size 983 bp (with MP144) |
| AJ38 | ACGTCCTCTCCCATGCTTGA | *mga2* KO checking primer, downstream, rev, product size 967 (with MP33) |
| MaP90 | GTATCGTCTTGCTCGGTT | ChIP-qPCR *atf1* (fwd) |
| MaP91 | CCACACTTCCACCTGTTT | ChIP-qPCR *atf1* (rev) |
| PD03 | GAATTACCAACGTCATATTTGC | ChIP-qPCR *ctt1* (fwd) |
| PD04 | ACTACGATAGGCTGTAGAAGA | ChIP-qPCR *ctt1* (rev) |
| MaP96 | TTGCTACAGGAAGAGGAAG | ChIP-qPCR *cut6* (fwd) |
| MaP97 | TAGAAAAGTTGGATGCGTG | ChIP-qPCR *cut6* (rev) |
| PD07 | GCGTCACTCGTCACATTA | ChIP-qPCR *pyp2* (fwd) |
| PD08 | TGCTAAGCGACCGTTTATT | ChIP-qPCR *pyp2* (rev) |
| MP88 | AGCTGCTAGACACCTTCAAA | ChIP-qPCR intergenic locus P4 (fwd) |
| MP89 | CCTACGGTCAAGAGAAAACT | ChIP-qPCR intergenic locus P4 (rev) |
| MP33 | GCGCACGTCAAGACTGTC | universal KO checking primer (*kanMX6/natMX6* cassette), downstream, fwd |
| MP144 | GTCGTTAGAACGCGGCTACA | universal KO checking primer, upstream, rev |
| MP150 | AGGGATCGAAAGACATCCGC | *cbf11* KO checking primer, upstream, fwd, product size 953 bp (with MP144) |
| MP151 | GCTTGTACACACGGCCTTCAA | *cbf11* KO checking primer, downstream, rev, product size 823 (with MP33) |
| MaP169 | AAAAAGCTAAATGATGCT | TAP tag |
| MP28 | GATACAGCAACTCCTCCCG | *cbf11* KO checking primer, outer genomic sequence, rev |
| MP53 | AGGTTAATACGCAATGG | *cbf11* ORF, to amplify region around DBM (with MP54), rev |
| MP54 | TTAGTATTGTCTCCAAACC | *cbf11* ORF, to amplify region around DBM (with MP53), fwd |
| AJ11 | GCGCAGCTCAGTTTTAGAGCTAGAAATAGCAAGTTAAAATAA | CRISPR/Cas9, first half of sgRNA against *natMX6* (TEF promoter), overlap into pMZ374, fwd |
| AJ12 | ACGTCAAGACTTCTTCGGTACAGGTTATGTTTTTTGGCAACA | CRISPR/Cas9, second half of sgRNA against *natMX6* (TEF promoter), overlap into pMZ374, rev |
| AJ29 | cctttataacGTTTTAGAGCTAGAAATAGCAAGTTAAAATAA | CRISPR/Cas9, first half of sgRNA against *cbf11* ORF (next to desired DBM), overlap into pMZ374, fwd |
| AJ30 | ctgacgactgTTCTTCGGTACAGGTTATGTTTTTTGGCAACA | CRISPR/Cas9, second half of sgRNA against *cbf11* ORF (next to desired DBM), overlap into pMZ374, rev |
| JT68 | CAAGGCCAAGGAATCCATTA | RT-qPCR *hsp9* ORF (fwd) |
| JT69 | GAGCCTTGTCATGAGCCTCT | RT-qPCR *hsp9* ORF (rev) |
| JT70 | TTCATGCGGTTTGACTTCAG | RT-qPCR *srx1* ORF (fwd) |
| JT71 | CCCCCAAAGGCAAAATAATA | RT-qPCR *srx1* ORF (rev) |

**Table B – List of plasmids**

| **ID** | **Vector** | **Experiment/use** | **Source** |
| --- | --- | --- | --- |
| pMP90 | pCloneNAT1 | KO vector for *cbf11* (*kanR* cassette) | This study |
| pMP91 | pCloneNAT1 | KO vector for *cbf11* (*natR* cassette) | Ref. 1 |
| pMP148 | pCloneNAT1 | KO vector for *ssp2* (*natR* cassette) | This study |
| pMP161 | pCloneNAT1 | KO vector for *mga2* (*natR* cassette) | This study |
| pMP134 | pMZ374 | Cas9/sgRNA_TEFp; Cas9, sgRNA against *natMX6* (TEF promoter) | This study |
| pMP153 | pMZ374 | Cas9/sgRNA_cbf11; Cas9, sgRNA against *cbf11* ORF (next to desired DBM) | This study |
| pMZ374 |  | Cas9/sgRNA_empty; Cas9, no sgRNA | Addgene plasmid # 59896 / Mikel Zaratiegui |
| pMaP11 | pUC19 | *cbf11DBM-TAP*, template for homologous recombination | This study |
| pMaP27 | pUC19 | *cbf11-TAP*, template for homologous recombination | This study |
| p407.C169S | pREP | fluorescence measurement of intracellular H_2_O_2_ | Ref. 2 |

**Table C – List of strains**

| **ID** | **Genotype** | **Source** | **Figure #** |
| --- | --- | --- | --- |
| JB32 | *h+* | Lab stock | Fig 1B bottom panel, Fig 1C, Fig 2B-E, Fig 3E, Fig 4A, Fig 4B, Fig 4D, Fig 4E, Fig 5A-F, Fig S1E, Fig S2, Fig S4, Fig S5 |
| MP114 | *h+ cbf11Δ::natR* | Ref. 1 | Fig 1C, Fig 2C-E |
| MP21 | *h+ cbf12Δ::natR* | Ref. 1 | Fig 1C, Fig 4A |
| MP22 | *h+ cbf11Δ::kanR* | Ref. 1 | Fig 4A, Fig4B |
| MP25 | *h+ cbf11Δ::kanR cbf12Δ::natR* | Ref. 1 | Fig 1C |
| JB146 | *h- atf1∆::ura4 ura4-D18* | Lab stock | Fig 2B, Fig 2C |
| MP367 | *h- ura4-D18 atf1∆::ura4 cbf11Δ::natR* | This study | Fig 2B, Fig 2C |
| MP44 | *h+ cbf11Δ::kanR* | Ref. 1 | Fig 1B bottom panel, Fig 2B, Fig 3E, Fig 4E, Fig 5A, Fig 5B, Fig S2, Fig S4 |
| JB147 | *h- pap1Δ::ura4 ura4-D18* | Lab stock | Fig 2D, Fig 2E |
| MP368 | *h- pap1Δ::ura4 cbf11Δ::natR ura4-D18* | This study | Fig 2D, Fig 2E |
| JB149 | *h- sty1∆::ura4 ura4-D18* | Lab stock | Fig 2B, Fig S2 |
| MP809 | *h+ sty1.T97A ura4-D18* | Ref. 3 | Fig 2A, Fig S2 |
| MP810 | *h+ sty1.T97A ura4-D18 Δcbf11::natR* | This study | Fig 2A, Fig S2 |
| IC132 | *h+ pap1-GFP::kanMX6* | Ref. 4 | Fig 3C |
| MP705 | *h+ cbf11-ctap4* | This study | Fig 3E |
| MP712 | *h+ cbf11DBM-ctap4* | This study | Fig 3E, Fig 4D, Fig 4E |
| MP815 | *h+ mga2Δ::natR* | This study | Fig 4D, Fig 4E |
| MP636 | *h- Pcut6MUT* | This study | Fig 4D, Fig 4E |
| MP218 | *h+ cut6-621* | Ref. 5 | Fig 4D, Fig 4E |
| MP19 | *h+ cbf11-ctap4::natR* | Ref. 1 | Fig 3D |
| MP15 | *h- cbf11-ctap4::natR ura4-D18 leu1-32 ade6-M216* | Ref. 1 |  |
| MaP70 | *h- cbf11-3HA::natMX6 ura4-D18 leu1-32 ade6-M216* | This study |  |
| MP670 | *h- cbf11DBM-3HA::nonfunctional_natMX6 ura4-D18 leu1? ade6?* | This study |  |
| MP865 (MS112) | *h+ gcn5::kanMX6* | Ref. 6 | Fig 5E |
| MP879 | *h+ gcn5::kanMX6 Δcbf11::natR* | This study | Fig 5E |
| MP880 | *h+ gcn5::kanMX6 Δcbf11::natR* | This study | Fig 5E |
| 972 | *h-* | Lab stock | Fig 1B top panel, Fig 1F, Fig 3A |
| AV18 | *h- sty1Δ::kanMX6* | Ref. 7 | Fig 3A |
| CS38 | *h- atf1-HA:natMX6* | Ref. 8 | Fig 3B |
| CS89 | *h- cbf11Δ::kanMX6* | This study | Fig 1B top panel, Fig 3A |
| CS95 | *h? cbf11Δ::kanMX6 atf1Δ::natMX6* | This study | Fig S1B |
| CS99 | *h+ cbf11Δ::kanMX6* | This study | Fig S1B |
| CS102 | *h- cbf11-HA:kanMX6* | This study | Fig S3A |
| CS110 | *h- cbf12Δ::kanMX6* | This study | Fig 1F |
| CS129 | *h+ cbf11-TAP:kanMX6* | This study | Fig S3B |
| CS142 | *h? atf1-HA:natMX6 cbf11::kanMX6* | This study | Fig 3B |
| MS98 | *h- atf1Δ::natMX6* | Ref. 9 | Fig S1A |
| HM123 | *h- leu1-32* | Lab stock |  |
| MP895 (IV16) | *h+ elp3::natMX6* | Ref. 9 | Fig S5B |
| MP904 (ENY2778) | *h- mst1-L344S-5FLAG:kanMX6 leu1-32 ura4-D18* | Ref. 10 | Fig 5D |
| MP905 (IV65) | *h- hat1::kanMX6* | Lab stock | Fig S5B |
| MP907 (JA2344) | *h+ mst2::kanR* | Lab stock | Fig S5C |
| MP908 (JA2242) | *h- rtt109:kanR* | Lab stock | Fig S5C |
| MP925 | *h+ elp3::natMX6 cbf11::kanR* | This study | Fig S5B |
| MP927 | *h- hat1::kanMX6 cbf11::natR* | This study | Fig S5B |
| MP948 | *h- mst1-L3444S-5FLAG:kanMX6 leu1-32 ura4-D18 cbf11::natR* | This study | Fig 5D |
| MP950 | *h+ mst2::kanR cbf11::natR* | This study | Fig S5C |
| MP951 | *h- rtt109:kanR cbf11::natR* | This study | Fig S5C |
| MP550 | *h+s natMX6-Padh1-3HA:cut6+* | Ref. 11 | Fig 5B, Fig 5C |
| MP555 | *h+ Δcbf11::kanR natMX6-Padh1-3HA:cut6+* | Ref. 11 | Fig 5B, Fig 5C |
| MP635 | *h+ Δssp2::natR* | This study | Fig S5A |
| MP855 | *h+ Δssp2::natR Δcbf11::hygR* | This study | Fig S5A |
| MP606 | *h- ppc1-537* | Ref. 12 | Fig S4B |

**SUPPLEMENTARY REFERENCES**

1. Převorovský M, Oravcová M, Tvarůžková J, Zach R, Folk P, Půta F, *et al*. (2015) Fission yeast CSL transcription factors: Mapping their target genes and biological roles. *PLoS One*, **10**, 1–23.

2. Carmona M, de Cubas L, Bautista E, Moral-Blanch M, Medraño-Fernández I, Sitia R, *et al*. (2019) Monitoring cytosolic H2O2 fluctuations arising from altered plasma membrane gradients or from mitochondrial activity. *Nat. Commun.*, **10**, 1–13.

3. Zuin A, Carmona M, Morales-Ivorra I, Gabrielli N, Vivancos AP, Ayté J, *et al*. (2010) Lifespan extension by calorie restriction relies on the Sty1 MAP kinase stress pathway. *EMBO J.*, **29**, 981–991.

4. Marte L, Boronat S, Barrios R, Barcons-Simon A, Bolognesi B, Cabrera M, *et al*. (2022) Expression of Huntingtin and TDP-43 Derivatives in Fission Yeast Can Cause Both Beneficial and Toxic Effects. Int. J. Mol. Sci. 7, 3950.

5. Saitoh S, Takahashi K, Nabeshima K, Yamashita Y, Nakaseko Y, Hirata A, *et al*. (1996) Aberrant mitosis in fission yeast mutants defective in fatty acid synthetase and acetyl CoA carboxylase. *J. Cell Biol.*, **134**, 949–961.

6. Sansó M, Vargas-Pérez I, Quintales L, Antequera F, Ayté J and Hidalgo E. (2011) Gcn5 facilitates Pol II progression, rather than recruitment to nucleosome-depleted stress promoters, in *Schizosaccharomyces pombe*. *Nucleic Acids Res.*, **39**, 6369–6379.

7. Zuin A, Vivancos AP, Sansó M, Takatsume Y, Ayté J, Inoue Y, *et al*. (2005) The glycolytic metabolite methylglyoxal activates Pap1 and Sty1 stress responses in *Schizosaccharomyces pombe*. *J. Biol. Chem.*, **280**, 36708– 36713.

8. Salat-Canela C, Paulo E, Sánchez-Mir L, Carmona M, Ayté J, Oliva B, *et al*. (2017) Deciphering the role of the signal- and Sty1 kinase-dependent phosphorylation of the stress-responsive transcription factor Atf1 on gene activation. *J. Biol. Chem.*, **292**, 13635–13644.

9. Fernández-Vázquez J, Vargas-Pérez I, Sansó M, Buhne K, Carmona M, Paulo E, *et al.* (2013) Modification of tRNA LysUUU by Elongator Is Essential for Efficient Translation of Stress mRNAs. *PLoS Genet.*, **9**.

10. Garabedian MV, Noguchi C, Ziegler MA, Das MM, Singh T, Harper LJ, *et al.* (2012) The double-bromodomain proteins Bdf1 and Bdf2 modulate chromatin structure to regulate S-phase stress response in *Schizosaccharomyces pombe. Genetics*, **190,** 487-500.

11. Převorovský M, Oravcová M, Zach R, Jordáková A, Bähler J, Půta F, *et al*. (2016) CSL protein regulates transcription of genes required to prevent catastrophic mitosis in fission yeast. *Cell Cycle*, **15,** 3082-3093.

12. Nakamura T, Pluskal T, Nakaseko Y and Yanagida M. (2012) Impaired coenzyme A synthesis in fission yeast causes defective mitosis, quiescence-exit failure, histone hypoacetylation and fragile DNA. *Open Biol*., **2**, 120117.
