# Supplementary material for: Perturbed fatty-acid metabolism is linked to localized chromatin hyperacetylation, increased stress-response gene expression and resistance to oxidative stress
Source: PLoS Genet. 2023 Jan 10;19(1):e1010582. doi: 10.1371/journal.pgen.1010582 (PMC9870116; doi:10.1371/journal.pgen.1010582)
Supplement: Attachment — Submitted filename: PLoS_GENETICS_REVIEW_11_21_2022.pdf [file pgen.1010582.s009.pdf]

In this manuscript, the authors provide evidence that compromised fatty acid biosynthesis resulting mainly from loss of *cbf11*$^+$, which encodes a *S. pombe* CSL transcription factor causes resistance to H_2O_2, as well as upregulation of stress response genes that is dependent on the Sty1-Atf1-Pap1 pathways and chromatin hyperacetylation by the Gcn5 and Mst1 HATs. The authors also propose that the epigenetic regulation of stress response genes is potentially mediated by intracellular levels of acetyl-CoA. The link between inhibition of fatty acid synthesis and oxidative stress resistance has been previously observed in *S. cerevisiae*, but the regulatory mechanisms involved in this phenomenon have not be elucidated. The findings in this manuscript nicely fill in the gap of knowledge and are intriguing. This is a substantial body of work with state-of-the art functional genomics approaches and LC-MS analysis. The data is of high quality and the manuscript is well written and relatively easy to read. However, I have some concerns regarding the evidence supporting the conclusions and proposed model in the manuscript.

There is insufficient direct evidence supporting the model that H3K9 hyperacetylation at the promoters of oxidative stress genes in response to fatty acid synthesis inhibition is caused by increased levels of acetyl-CoA. Higher global levels of acetyl-CoA were not detected in the *cbf11* deletion and *cut6* hypomorph that run counter to this model. Although levels of acetyl-CoA could differ between cellular compartments, and thus provide a possible explanation of this model, there is no direct evidence from this manuscript that supports this. One possibility is that the hyperacetylation of histones in a *cbf11* deletion is not caused by higher (localized) acetyl-CoA levels as suggested, but rather enhanced activity of Gcn5/Mst1. Does Cbf11 directly repress Gcn5/Mst1 levels or activity since a physical interaction between Cbf11 and Gcn5 has been reported in the Pancaldi et al. (2012) paper. I also noticed that there is a putative Cbf11 consensus binding site within the *gcn5*$^+$ promoter suggesting that Cbf11 may regulate its transcription. The proposed model would require further experimentation and supportive data, such as the determination of expression and H3K9 acetylation levels of oxidative stress genes in unperturbed *fas1*, *fas2*, *ppc1-537* and *ssp2* null cells where fatty acid synthesis is compromised or acetyl-CoA levels are altered independently of Cbf11.

The observation that inhibition of fatty acid biosynthesis results in increased resistance to oxidative stress has been observed in S. cerevisiae *FAS1* (Matias et al. 2007; PMID 17936191) which was not cited in the manuscript. Whether Cbf11 directly represses one of several oxidative stress response pathways in unstressed cells or if compromised fatty acid biosynthesis elicits a general oxidative stress response independently of Cbf11 is not fully resolved and requires further investigation. The experiments performed in this paper are insufficient to make the firm conclusion that Cbf11 does not directly regulate the Sty1-Atf1-Pap1 pathway or another oxidative stress response pathway. Some important points that were not addressed in the paper include the following. Does Cbf11 regulate Pcr1 since it forms a heterodimer with Atf1 in the regulation of oxidative stress response genes? The Pancaldi et al. paper detected physical interactions of Cbf11 with Atf1, Pcr1 and Pap1 (as well as Prr1) by mass spectrometry. Could Cbf11 regulate these oxidative stress responsive transcription factors by repression of their activity or recruitment to promoters of target genes? Although the latter was investigated for Atf1, whether recruitment of Pcr1, Atf1 and Prr1 to their target gene promoters is altered in the *cbf11* deletion was not determined. For the Pap1-GFP experiments,

I think it is a worthwhile to examine nuclear localization in the *cbf11* deletion background. The nuclear translocation of Pap1 in the *cbf11* deletion may not be as obvious from H_2O_2 treatment, but a slight enhancement in nuclear localization could be revealed by quantitative measurements of the nuclear cytoplasmic ratio of Pap1-GFP. Also, does overexpression of *cbf11*$^+$ cause hypersensitive growth and repression of oxidative stress gene expression in response to H_2O_2 treatment?

Wild-type cells treated with cerulenin (inhibition of fatty acid synthesis independent of Cbf11) causes an induction of oxidative stress genes but is this regulation similar or distinct from Cbf11? Would wild-type cells exposed to cerulenin cause histone hyperacetylation at stress genes or is this response to compromised lipid biosynthesis only specific to Cbf11? Since cerulenin treatment appear to not involve Pap1 activation, are the other oxidative stress responsive transcription factors or Sty1 activated? Cerulenin treatment is transient and does not directly determine if compromised lipid biosynthesis independent of Cbf11 confers resistance to H_2O_2. It may be beneficial to determine if the *fas1* deletion confers resistance to H_2O_2, as in *S. cerevisiae* and if oxidative stress response genes/regulators are induced in this genetic background.
