# Supplementary material for: Perturbed fatty-acid metabolism is linked to localized chromatin hyperacetylation, increased stress-response gene expression and resistance to oxidative stress
Source: PLoS Genet. 2023 Jan 10;19(1):e1010582. doi: 10.1371/journal.pgen.1010582 (PMC9870116; doi:10.1371/journal.pgen.1010582)
Supplement: Attachment — Submitted filename: 127865_1_rebuttal_2477095_rj0ffb.docx [file pgen.1010582.s008.docx]

**Manuscript number:** RC-2022-01530

**Corresponding author(s):** Martin, Převorovský

## 1. General Statements

We would like to thank all reviewers for their time and effort, constructive criticism, and for helpful comments and suggestions. We have carefully addressed all points raised, amended the manuscript text where required, and added new results to support our conclusions. We now show that the Pap1 transcription factor is required for the moderately increased expression of some stress genes under conditions of decreased FA synthesis, but we also provide evidence that Pap1 is not hyperactivated under these conditions (similar to Sty1-Atf1). Next, we demonstrate that acute chemical inhibition of FA synthesis by cerulenin also triggers increased stress-gene expression (similar to genetic perturbations of FA synthesis). Finally, we show that overexpression of ACC/Cut6 sensitizes WT (and cbf11KO) cells to oxidative stress, further supporting a tight connection between FA synthesis and stress resistance. Based on all our data, we postulate a hypothesis that decreased FA synthesis results in increased activity of the Gcn5 and Mst1 HATs (possibly mediated by surplus acetyl-CoA) and hyperacetylation of a subset of stress gene promoters. This in turn creates a more transcription-competent chromatin environment and boosts the basal Sty1-Atf1 and Pap1-regulated transcription of these stress-response genes, resulting in increased resistance to oxidative stress.

## 2. Point-by-point description of the revisions

Reviewer #1 (Evidence, reproducibility and clarity (Required)):

This paper demonstrates a link between oxidative stress, lipid biosynthesis, and targeted histone acetylation in fission yeast. In mutant cells with defects in lipid synthesis (cbf11, mga2 lacking transcription factors, and cut6 lacking acetyl-CoA carboxylase), transcripts of a number of genes implicated in resistance to oxidative stress are increased. This is associated with higher levels of H3K9 acetylation and increased tolerance to oxidative stress. These effects are mediated through Sty1, a stress-activated MAP kinase and the transcription factor Atf1.

It is also shown that H3K9 acetylation levels in the promoter region and just downstream of the transcriptional start site are increased in cbf11 mutants (Fig. 5A).

By mutational analysis, the authors implicate the acetyl transferases Mst1 and Gcn5 in this transcriptional effect. Other related acetyl transferases, Hat1, Elp3, Mst2, Rtt109 have been ruled out as main contributors to the dysregulation in unstressed cbf11 mutants. That specific acetyl transferases have been shown to be required is a strength of the investigation.

Major comments:

The hypothesis is put forward in the manuscript that altered acetyl-CoA levels in cbf1 mutants would underlie the dysregulation of genes induced by oxidative stress. Histone acetyl transferases compete for acetyl-CoA with lipid biosynthesis, and so with increased demand for acetyl-CoA underacetylation in the concerned promoters would result - specifically at H3K9. These results do not directly support the hypothesis, on the other hand they are not sufficient to rule it out.

- Actually, we view this phenomenon the other way round: We primarily focus on exponentially growing cells, which have substantial demand for fatty acid (FA) production (= high acetyl-CoA consumption). So the level of promoter histone acetylation under these conditions is our baseline, or “normal” state. When FA production is decreased (cbf11 or cut6 mutants; inhibition of FA synthase by cerulenin…), stress gene promoters get *hyper*acetylated. We do not have any data on (or claims about) histone underacetylation compared to the baseline. Nevertheless, we now show that overexpression of Cut6/ACC results in decreased resistance to oxidative stress (Fig. 5C), which is compatible with the notion that increased acetyl-CoA consumption would result in insufficient histone acetylation at stress gene promoters during stress.

Acetyl-CoA levels were measured only in undisturbed cells, and the possibility remains that under oxidative stress there would be changes in acetyl-CoA pools that could explain this apparent contradiction - why did not the authors examine that?

- Under oxidative stress, the Sty1 stress MAPK is activated, leading to a massive Atf1-dependent transcription wave, which is also associated with increased SAGA-dependent H3K9 acetylation (PMID: 21515633). This well-studied cellular response, however, is not the main focus of our study. Rather, we found a novel connection between perturbed lipid metabolism and increased expression of stress genes in cells *not challenged* by oxidative stress (i.e. Sty1-Atf1 are not hyperactivated). This is why we only measured acetyl-CoA concentrations in untreated cells.

The authors argue that although the global acetyl-CoA levels are not increased, local concentrations might be altered in a way to permit higher H3K9 acetylation levels at selected promoters. Although a formal possibility, this is rather far-fetched as a small and freely diffusible molecule like acetyl-CoA should quickly equilibrate within one cellular compartment. I think that although the overall relationships that the authors have established between oxidative stress, H3K9 acetylation levels with increased expression, and lipid biosynthesis, are compelling, the role of acetyl-CoA concentrations is not clear and should be de-emphasized.

- Interestingly, acetyl-CoA production in the nucleus has been published by several studies (reviewed in PMID: 29174173), suggesting that local acetyl-CoA concentrations (microgradients) within the cell are functionally relevant. We agree that acetyl-CoA is a small molecule which, in theory, should diffuse quickly throughout the nucleocytoplasmic space. However, empirical evidence shows that the lipid synthesis in the cytosol and histone acetylation in the nucleus may not access a uniform nuclear-cytosolic pool of acetyl-CoA (PMID: 28099844, PMID: 28552616). This is related to the fact that the acetyl-CoA sink is large and acetyl-CoA may react with many proteins (i.e. any extra amounts will be consumed rapidly).
- Even though we provide strong evidence that HAT activity is critical for the crosstalk between FA synthesis and stress gene expression, we do agree that we have not conclusively established the role of acetyl-CoA in the process. However, we still feel that it is justified to point out acetyl-CoA is a “possible” mediator molecule for the crosstalk in the Results and Discussion sections.

Minor comments:

In many of the bar diagrams, only a borderline statistical significance is indicated (p ~ 0.05) despite seemingly large numerical differences between the means. In the legends it is stated that one-sided Mann-Whitney U tests were used. This is a non-parametric test with low power - would it not have been better to use a t test?

- We do agree that the non-parametric Mann-Whitney U test is rather conservative and, therefore, less sensitive for small sample sizes, such as n = 3. Our reason for using this particular test instead of the parametric t-test is that qPCR fold-change values come from a log-normal distribution, which is incompatible with t-test (requires normal distribution of data). Importantly, using conservative statistical testing does not invalidate our conclusions.

What do the error bars in the diagram show, SEM? If a non-parametric test is used, a parametric measure of variability is irrelevant.

- The error bars represent standard deviation (SD). We do not see an issue here as, in our opinion, the visual style of numeric data presentation is independent from any chosen statistical testing methods.

It would be helpful to the reader to indicate directly in the diagram panels what is actually shown, not just "fold change vs ..." In Fig. 1, 2, 4 D and 5 we see mRNA levels, in Fig. 3 chromatin IP.

- Done

Reviewer #1 (Significance (Required)):

The paper represents conceptual advances for our understanding of how stress responses, metabolism and transcriptional regulation are linked, although one of the links (acetyl-CoA levels in this case) is tenuous.

This manuscript belongs in a rich literature on stress responses on the gene expression level, mostly from studies in yeast. Potentially, it adds entirely new information on how cellular stress may be mechanistially linked to stress responses.

These results are potentially general and of broad interest to the biological community.

This reviewer is familiar with yeast genetics, stress responses, and quantification of gene expression.

Reviewer #2 (Evidence, reproducibility and clarity (Required)):

As more and more metabolic intermediates are found to also serve as co-factors for epigenetic modifications, it has been widely accepted that regulating the levels of these key metabolites can be an effective way to control nutrient related gene expression. Acetyl-CoA is one of those early examples. Increased acetyl-CoA was shown to promote local acetylation at growth genes (Mol Cell 2011 PMID: 21596309), and ACC deletion funnels more Acetyl-CoA towards histone acetylation reactions and causes global hyperacetylation (Ref 17). However, whether those increased metabolite/co-factor can exert signal-specific effects remains elusive. For instance, although increased acetyl-CoA stimulates the SAGA complex enzymatic activity, it is not clear whether it also causes SAGA to be targeted to new sites without external cues to induce new transcription factor binding. Does increased acetyl-CoA cause broad hyperacetylation at all inducible genes which are the primary targets for those HAT complexes?

In this manuscript, Princová et al. found that deletion of fatty acid synthesis transcriptional factors Cbf11 and Mga2 increases cell survival under H2O2 induced oxidative stress in S. pombe. They further showed that several stress-related genes increased upon Cbf11 deletion, and H3K9 acetylation at their promotor regions were elevated. They argued that FA-TF deletion may indirectly regulate stress-related genes potentially through influencing Acetyl-CoA level, although they failed to detect significant changes of global Acetyl-CoA levels. While it's interesting to see yet another example of metabolite-mediated gene expression regulation, the current manuscript only made incremental advance towards mechanistic principles of how these co-factors finetune specific gene expression program.

Specific comments:

1. This work showed convincingly that deletion of CBF11 or MGA2 leads to resistance to oxidative stress. However, it provides little mechanistic insight into how deletion of Cbf11 increased the expression of stress response genes and why some HATs are involved but others not (Figure EV5).

- We respectfully disagree with the notion that we only provide “little mechanistic insight” into the process whereby FA metabolism affects stress gene expression.
  - First, we show that not only deletion of cbf11, but also a very specific manipulation of the rate-limiting FA-producing enzyme (Cut6/ACC; Fig. 4D), or chemical inhibition of FA synthase by cerulenin (new Fig. 4F) all lead to increased stress gene expression. On the other hand, overproduction of Cut6/ACC results in decreased stress gene expression and lower resistance to ox. stress (new Fig. 5B-C). These findings clearly show the specific and tight mutual relationship between FA synthesis and expression of stress genes.
  - Second, we show that the DNA-binding activity of Cbf11 is critical for affecting stress gene expression levels, yet Cbf11 does not act as a stress gene repressor.
  - Third, we show that, compared to e.g. peroxide treatment, stress gene mRNA levels are only moderately increased upon downregulation of FA synthesis. So the situation can be called stress gene “derepression”. At the same time the major stress-response regulators (Sty1-Atf1, Fig. 2A-C; Pap1, new Fig. 2D-E) are required for the derepression, but, importantly, neither of them shows increased activation compared to unstressed WT cells (Fig. 3A-C). These data suggest a qualitative difference between the two phenomena (canonical stress response vs dysregulation of FA synthesis). Furthermore, they hint at an important role of the chromatin environment.
  - Fourth, we show that Gcn5/SAGA and Mst1, but not 4 other HATs, mediate the connection between FA metabolism and stress gene expression (Fig. 5D-E), and we show clear and specific H3K9 hyperacetylation of stress gene promoters in FA metabolism mutants (Fig. 5A), arguing that this is not a general acetylome issue.
  - Fifth, we show that the stress genes affected by changes in FA metabolism show unusually high nucleosome (H3) occupancy in their transcribed regions (even in unperturbed WT cells; Fig. 5A bottom panels), which could dictate the observed specificity in regulation.

While we agree that our understanding is not yet complete, we have already described many mechanistic aspects of the link between FA metabolism and stress gene expression.

2. Although in Cbf11 deletion cells, increased resistance to H2O2 is relied upon the Sty1/Atf1 pathway, the authors did not establish a link between lipid synthesis and Atf1 activity because Cbf11 deletion does not affect the phosphorylation of Atf1.

- Sty1 and/or Atf1 show non-zero activity even in normal, healthy, unstressed cells. Importantly, Atf1 is bound to many target promoters even in the absence of stress (Fig. 3B; PMID: 20661279, PMID: 28652406). Moreover, Sty1 is actually needed for orderly cell cycle progression (sty1KO cells are elongated, a result of postponed mitotic entry; e.g. PMID:7501024), which we now mention in the Introduction and Discussion. Our point is that Sty1-Atf1 are not hyperactivated under normal conditions - this only happens during major stress insults. Thus, in unstressed cbf11KO cells, stress gene promoters are hyperacetylated, which may facilitate their (Sty1-Atf1 and Pap1-dependent) transcription, without the need for hyperactivation of the stress response regulators. Such increased transcriptional competence of stress promoters is consistent with our findings that upon peroxide treatment stress gene mRNA levels in cbf11KO exceed those in WT (Fig. 1B). We have amended the corresponding section of the Discussion to more clearly explain our conclusions and hypotheses.

3. Cbf11 deletion causes elevated H3K9 acetylation at the promotor regions of a number of stress respond genes, the author did not mention whether demonstrate how lipid synthesis defect causes the hyperacetylation at these promoters.

- As discussed in our manuscript, we suggest that following downregulation of FA synthesis, the surplus acetyl-CoA is used by Gcn5 and Mst1 HATs to hyperacetylate stress gene promoters.

3. As all lipid-metabolism mutants show increased stress response, it would helpful to examine whether H2O2 induction of WT cells influence lipid synthesis, thus establish physiological links between FA synthesis and stress response.

- We now mention in the Discussion section that, curiously, cut6/ACC mRNA levels are downregulated upon peroxide treatment. However, the significance of this finding is unclear as FA metabolism is strongly regulated at the post-translational level (PMID: 12529438). Unfortunately, we are not in a position to measure changes in metabolic fluxes upon stress. In any case, we believe that such experiments would be outside the scope of the current study.

Beside, fatty acid may be beneficial to fight oxidative stress because they maintain the integrity of cell membrane. What is the potential effect of CBF11 deletion in this aspect? The author may want to discuss it.

- The reviewer suggests that higher production of FA would result in higher resistance to oxidative stress. However, our data do not indicate this - we show that under low FA synthesis the stress resistance is actually higher. Nevertheless, we acknowledge in the Discussion that the scenario suggested by the reviewer can occur, for example, in cancer cells which become more resistant to oxidative stress following increased lipid biosynthesis/storage.

4. Since H2O2 treatment also causes change in glucose metabolism including upregulation of glucose transporter Ght5 (PMID: 30782292), it would be enlightening to see if there is a crosstalk between the lipid and glucose metabolisms. Does Ght5 expression increase upon H2O2 treatment in CBF11 deletion strain?

- While the topic is interesting, we strongly believe that the relationship between glucose metabolism and stress gene expression is outside the scope of this study.
- According to our data used in Fig. 4A, ght5 expression in cbf11KO at 60 min after 0.74 mM H2O2 treatment is downregulated 3-fold.

5 Different H2O2 concentration causes different stress response in pombe: Pap1 and Sty1 mediate responses for low and high H2O2, respectively. For fully activated Sty1 response, the concentration of H2O2, needs to reach 1mM (PMID: 17043891). In this study, the H2O2 concentration ranges from 0.5-1.5mM and Pap1 regulated Ctt1 does show increase upon H2O2 treatment. To test if suppressed lipid synthesis facilitates Sty1 dependent activation, it would be helpful to examine the activation of Pap1 (its nuclear translocation) to eliminate other influences.

- We agree with the reviewer. We have now included data on the role of Pap1 in the crosstalk between lipid metabolism and stress gene expression. We show that Pap1 is required for increased expression of gst2 and ctt1 in untreated cbf11KO cells (Fig. 2D). We note that ctt1 is coregulated by both Pap1 and Atf1 (Fig. 2B, D). Also, Pap1 is partially required for H2O2 resistance of cbf11KO cells (Fig. 2E). Importantly, similar to Sty1-Atf, Pap1 is not hyperactivated (no nuclear accumulation) by 10 or 60 min of cerulenin treatment (Fig. 3C), while stress gene expression is upregulated at 60 min in cerulenin (Fig. 4F) and keeps increasing after 120 min (data not shown). These data collectively support our hypothesis that upon decreased FA synthesis, stress gene promoters become more transcription-competent without the requirement for hyperactivation of the corresponding stress gene regulators.

Reviewer #2 (Significance (Required)):

see above

Reviewer #3 (Evidence, reproducibility and clarity (Required)):

This study examines the intriguing phenomenon that perturbation of fatty acid biosynthesis induces expression of stress-response genes by increased intracellular levels of acetyl-CoA and hyperacetylation of histones at the promoters of these genes. Loss of the CSL transcription factor Cbf11 results in induced expression of a subset of stress-response genes in unperturbed conditions and resistance to H2O2. These stress-response genes are not direct targets of Cbf11, but their upregulation is dependent on the Sty1-Atf1 pathway. Similar effects in upregulation of stress-response genes were observed in the cut6 hypomorph and mga2 deletion strain, however no change in global levels of acetyl-Co-A in the former as well as in the cbf11 deletion was detected. The upregulated stress-response genes appear to be linked to increased H3K9 acetylation in their promoters and dependent on the Gcn5 and Mst1 HATs.

The authors present good supportive evidence linking fatty acid biosynthesis to epigenetic regulation of stress response genes potentially mediated by intracellular levels of acetyl-CoA. This is an exciting area and the fission yeast model system is ideal to elucidate the molecular mechanisms behind this process. This is a substantial body of work with state-of-the art functional genomics approaches and LC-MS analysis. The data is of high quality and the manuscript is well written and relatively easy to read. Below are my comments for the manuscript.

It was determined that increased expression of stress-response genes in the cbf11 deletion is dependent on the presence of Sty1, and partially dependent on Atf1. How about Pap1 (or Prr1) - would this transcription factor that is also regulated by Sty1 be involved in the upregulation of the stress-response genes in the cbf11 deletion? Activation of Sty1 and Atf1 by phosphorylation was not observed in unperturbed cbf11 deletion cells which would be expected in the proposed model. This discrepancy was not well explained. Could activation of Sty1/Atf1/Pap1 in unperturbed cbf11 cells be assayed in a different way such as nuclear localization?

- As these concerns were also raised by Reviewer 2, to avoid duplication, we kindly ask you to read our detailed responses above. Briefly, we have now included new data clarifying the role of Pap1 in the increased expression of selected stress genes in cbf11KO cells (or when FA synthesis is chemically inhibited) - comment #5 of Reviewer 2 above. Also, we explain why Sty1-Atf1 and/or Pap1 hyperactivation (i.e. above their activity level in untreated WT) is actually not needed in order for decreased FA synthesis to trigger a mild/moderate increase in stress gene expression - comment #2 of Reviewer 2 above. We have now also clarified this issue in the Discussion section.
- As for the use of alternative methods for measuring the activation status of Sty1-Atf, we have already provided data from multiple independent and very sensitive methods (western blot, ChIP-qPCR; Fig. 3A-B). Also, it is questionable whether microscopy would be more sensitive than our current methods. Moreover, our H2O2-sensitive reporter does not indicate an increasingly oxidative environment inside cbf11KO cells, quite on the contrary (Fig. 1D).

It would strengthen the model that perturbation of fatty biosynthesis induces expression of stress-response genes and H2O2 resistance if more mutant strains other than cut6 and two of its known regulators were tested. Does the proposed model apply to any deficiency in fatty acid synthesis in general or only those that result in increased levels of acetyl-CoA? For example, would deletion strains of fas1, fas2, lsd90, lcf1, lcf2 or the4 show the same stress response as cut6, mga2, and cbf11 mutants?

- The roles of lsd90, lcf1, lcf2 and the4 have been only poorly characterized so far, making it potentially difficult to interpret any stress-related phenotypes of these mutants. However, the role of the fatty acid synthase Fas1/Fas2 complex in FA production is well established. We have therefore inhibited FAS using cerulenin and found that this treatment also leads to increased stress gene expression (Fig. 5F), without causing Pap1 hyperactivation (Fig. 3C). Importantly, fas1/fas2 are not Cbf11 target genes, and FAS inhibition by cerulenin represents an acute intervention, very different from the long-term effects in cbf11/mga2/cut6 mutants.

Also, does overexpression of cut6+ confer sensitivity to H2O2?

- Yes, our new data show that ~2-fold overexpression of cut6 both partially abolished the derepression of stress genes in cbf11KO cells (Fig. 5B), and increased sensitivity to H2O2 of WT cells (new Fig. 5C).

The authors hypothesize that induced expression of stress-response genes in the cbf11 deletion and cut6 hypomorph is due to H3K9 hyperacetylation because of increased acetyl-CoA abundance in the cell. However, LC-MS analysis showed no change in global abundance of acetyl-CoA in the cbf11 deletion and cut6 hypomorph although differential levels of acetyl-CoA in the nucleus relative to the rest of the cell cannot be ruled out. The authors mentioned that ppc1-537 and ssp2 null are known to have lower abundance of acetyl-CoA and the latter could suppress the cbf11 deletion-induced gene expression for two of three genes tested by qPCR. Can ppc1-537 also suppress the cbf11 deletion-induced gene expression? Are ppc1-537 and the ssp2 null sensitive to H2O2?

- The ppc1-537 mutant is sick and has a growth defect, making it difficult to interpret any findings regarding its survival/resistance phenotype (see a similar issue with the cut6-621 mutant in Fig. 4E). Ssp2/AMPK has a pleiotropic role in the cell and its activity is actually controlled by Sty1-Atf1 under some stress conditions (PMID: 28515144) and the ssp2KO is resistant to osmotic stress (PMID: 28600551). All this makes it potentially difficult to derive reliable conclusions about ppc1 and ssp2. However, our current data on cut6 (ts hypomorph, Pcut6MUT, overexpression) and FAS/cerulenin are derived from precisely targeted and specific interventions, and support the proposed connection between FA synthesis and stress gene expression, and are consistent with the suggested role of acetyl-CoA (and its microgradients) in mediating the connection.

I think Rtt109 is H3K56 specific.

- Indeed, H3K56 is the characterized specificity of Rtt109, and we indicate this explicitly in the manuscript. We wanted to make our HAT screen comprehensive since we could not presume which histone or even non-histone acetylation target(s) is involved in lipid metabolism-mediated stress gene expression. Even though we have observed increased H3K9ac (Gcn5/SAGA target), other modifications are likely involved since Mst1 affects stress gene expression in lipid mutants, but Mst1 is not known to target H3K9.

Reviewer #3 (Significance (Required)):

The authors present good supportive evidence linking fatty acid biosynthesis to epigenetic regulation of stress response genes potentially mediated by intracellular levels of acetyl-CoA. This is an exciting area and not all the molecular details have been elucidated in this process. S. pombe is ideal to study this fundamental process and discoveries would be applicable to other eukaryotic study organisms.

My expertise is in eukaryotic gene regulation, molecular genetics and functional genomics, so I am quite qualified to critically review this paper.
