# Supplementary material for: Perturbed fatty-acid metabolism is linked to localized chromatin hyperacetylation, increased stress-response gene expression and resistance to oxidative stress
Source: PLoS Genet. 2023 Jan 10;19(1):e1010582. doi: 10.1371/journal.pgen.1010582 (PMC9870116; doi:10.1371/journal.pgen.1010582)
Supplement: Attachment — Submitted filename: Response to Reviewers PLoS Genetics.docx [file pgen.1010582.s010.docx]

We would like to thank both reviewers for evaluating our manuscript. Our responses to your comments and suggestions are given below in blue.

Since our proposition that changes in acetyl-CoA availability could mediate a specific crosstalk between FA metabolism and stress gene expression has repeatedly received criticism during the lifetime of this manuscript, we have eventually decided to cut back on our speculations in this direction. While we still discuss known and potential roles of this important metabolite, we clearly state that we do not provide any mechanistic details or explanations supporting acetyl-CoA as a specificity mediator. Accordingly, we have removed acetyl-CoA from our final model (Fig. 5F), and we now also discuss alternative ways how specificity in the link between FA metabolism and stress gene expression might be achieved.

Reviewer's Responses to Questions

**Comments to the Authors:**

**Please note here if the review is uploaded as an attachment.**

**Reviewer #1:** In the revised version, the authors present new data, and respond to the criticism. They provide evidence that Pap1 is needed for stress gene activation when lipid metabolism is perturbed. Importantly, a new experiment has been added showing that overexpression of cut6+ does make cells more sensitive to H2O2 (Fig. 5C), further demonstrating that histone acetylation is important for oxidative stress resistance.

My main concern was the authors’ claim that altered acetyl-CoA availability during oxidative stress or defects in lipid metabolism was the cause of altered histone acetylation at promoters, which in turn affects transcription.

The first problem is that the authors have measured and not found any change in global acetyl-CoA levels. They then reason that local availability of this molecule at certain promoters could still be altered.

Towards the end of the Results section, we now mention a theoretical possibility that the apparent lack of changes in acetyl-CoA levels in the Pcut6mut and cbf11KO cells (Fig S4B) might be due to rapid consumption of any surplus acetyl-CoA by HATs.

The problem then becomes to explain how this could selectively affect certain promoters. The authors argue that even if acetyl-CoA is a small and freely diffusible molecule which should quickly redistribute within a certain cellular compartment, there is evidence for separate pools of nuclear and cytoplasmic acetyl-CoA pools. In my view, this is not sufficient evidence. We would have to postulate a mechanism for highly localized subpools around certain promoters. One way I could imagine this to happen would be by phase separation, forming liquid condensates that slow down diffusion, and I am not aware of evidence for such condensates here. One could also imagine acetyl-CoA synthesis physically linked to e.g. chromosome domains or promoters. This remains speculation however.

We accept your criticism.

As stated at the very beginning of our responses, we have substantially limited our speculative claims regarding the role of acetyl-CoA in regulating the expression of specific (stress-responsive) genes.

Nevertheless, I find that overall, there is sufficient interesting new data in the manuscript to warrant publication, even if I remain unconvinced about the proposed mechanism.

Thank you for your support.

I would like to see the authors further develop their arguments about how acetyl-CoA availability could be locally restricted (to very small volumes, certainly not the whole nucleus) in the cell to explain their observations. Are there alternative possibilities?

We now also discuss whether Cbf11 could directly regulate (repress) the Gcn5 and Mst1 HATs (we find this unlikely). Furthermore, we discuss that HAT specificity can be dictated by HAT complex composition (eg, SAGA is recruited by different mechanisms to different genes: While the Tra1 subunit of SAGA is crucial for mediating the interaction with specific transcription factors at some loci, it is dispensable at others; PMID 21642955).

**Reviewer #2**: Reviewer's comments were uploaded as an attachment.

In this manuscript, the authors provide evidence that compromised fatty acid biosynthesis resulting mainly from loss of cbf11+ , which encodes a S. pombe CSL transcription factor causes resistance to H2O2, as well as upregulation of stress response genes that is dependent on the Sty1-Atf1-Pap1 pathways and chromatin hyperacetylation by the Gcn5 and Mst1 HATs. The authors also propose that the epigenetic regulation of stress response genes is potentially mediated by intracellular levels of acetyl-CoA. The link between inhibition of fatty acid synthesis and oxidative stress resistance has been previously observed in S. cerevisiae, but the regulatory mechanisms involved in this phenomenon have not be elucidated. The findings in this manuscript nicely fill in the gap of knowledge and are intriguing. This is a substantial body of work with state-of-the art functional genomics approaches and LC-MS analysis. The data is of high quality and the manuscript is well written and relatively easy to read. However, I have some concerns regarding the evidence supporting the conclusions and proposed model in the manuscript.

There is insufficient direct evidence supporting the model that H3K9 hyperacetylation at the promoters of oxidative stress genes in response to fatty acid synthesis inhibition is caused by increased levels of acetyl-CoA. Higher global levels of acetyl-CoA were not detected in the cbf11 deletion and cut6 hypomorph that run counter to this model. Although levels of acetyl- CoA could differ between cellular compartments, and thus provide a possible explanation of this model, there is no direct evidence from this manuscript that supports this.

We would like to point out that while our MS data on acetyl-CoA levels do not directly support the proposed model, they do not falsify it either. The measurement of acetyl-CoA is technically challenging and potentially small/transient changes are difficult to capture. However, we do accept your criticism and, as stated at the very beginning of our responses, we have largely limited our speculative claims regarding the role of acetyl-CoA in regulating specific (stress-responsive) genes.

One possibility is that the hyperacetylation of histones in a cbf11 deletion is not caused by higher (localized) acetyl- CoA levels as suggested, but rather enhanced activity of Gcn5/Mst1. Does Cbf11 directly repress Gcn5/Mst1 levels or activity since a physical interaction between Cbf11 and Gcn5 has been reported in the Pancaldi et al. (2012) paper. I also noticed that there is a putative Cbf11 consensus binding site within the gcn5+ promoter suggesting that Cbf11 may regulate its transcription.

Thank you for this suggestion. We now discuss the possibility of Cbf11 directly regulating (repressing) Gcn5/Mst1 activities in the Discussion. We believe that this scenario is unlikely for the following reasons:

- We did not see any Cbf11 binding to either gcn5 or mst1 promoters (PMID: 26366556)
- mst1 and gcn5 transcript levels are not upregulated in cbf11KO (PMID: 26366556)
- The Pancaldi paper that you mention has one important limitation - no DNase treatment was performed during Cbf11 pulldown, so any detected interactions can be indirect (DNA-mediated) or spurious (PMID: 22540037)

The proposed model would require further experimentation and supportive data, such as the determination of expression and H3K9 acetylation levels of oxidative stress genes in unperturbed fas1, fas2, ppc1-537 and ssp2 null cells where fatty acid synthesis is compromised or acetyl-CoA levels are altered independently of Cbf11.

Our data showing increased promoter H3K9 acetylation at a subset of stress genes indeed do not prove that this was caused by altered acetyl-CoA levels, or by Cbf11 manipulation. It is merely a correlation. Nevertheless, we show that specific stress gene expression is induced in cerulenin-treated cells (FAS inhibition; Fig. 4) and lipid metabolism mutants (cut6-621, Pcut6MUT, mga2; Fig. 4D). Moreover, we also show that stress gene upregulation in cbf11KO is diminished by overexpression of cut6 (Fig. 5B) or deletion of ssp2 (Fig. S5A), and by mutations of the Gcn5 and Mst1 HATs (Fig. 5D, E). We believe that we have thereby shown convincingly that decreased FA synthesis leads to increased stress gene expression and higher cellular resistance to oxidative stress. In our opinion, the validity of this main message of our paper does not depend on any particular role of histone acetylation (even though we speculate that acetylation is implicated, given the importance of HATs).

The observation that inhibition of fatty acid biosynthesis results in increased resistance to oxidative stress has been observed in S. cerevisiae FAS1 (Matias et al. 2007; PMID 17936191) which was not cited in the manuscript.

Thank you for directing us to this interesting reference. We now mention the Matias et al. 2007 paper in our manuscript (Results section). These authors suggested that the increased resistance to oxidative stress of fas1KO budding yeast cells was due to altered membrane lipid composition and potentially lower membrane permeability for H2O2. However, our data rather show that the increased ox. stress resistance of fission yeast lipid metabolism mutants is due to changes in stress gene expression. Nevertheless, we acknowledge that both proposed mechanisms may apply.

Whether Cbf11 directly represses one of several oxidative stress response pathways in unstressed cells or if compromised fatty acid biosynthesis elicits a general oxidative stress response independently of Cbf11 is not fully resolved and requires further investigation. The experiments performed in this paper are insufficient to make the firm conclusion that Cbf11 does not directly regulate the Sty1-Atf1-Pap1 pathway or another oxidative stress response pathway. Some important points that were not addressed in the paper include the following. Does Cbf11 regulate Pcr1 since it forms a heterodimer with Atf1 in the regulation of oxidative stress response genes? The Pancaldi et al. paper detected physical interactions of Cbf11 with Atf1, Pcr1 and Pap1 (as well as Prr1) by mass spectrometry. Could Cbf11 regulate these oxidative stress responsive transcription factors by repression of their activity or recruitment to promoters of target genes? Although the latter was investigated for Atf1, whether recruitment of Pcr1, Atf1 and Prr1 to their target gene promoters is altered in the cbf11 deletion was not determined.

For the Pap1-GFP experiments, I think it is a worthwhile to examine nuclear localization in the cbf11 deletion background. The nuclear translocation of Pap1 in the cbf11 deletion may not be as obvious from H2O2 treatment, but a slight enhancement in nuclear localization could be revealed by quantitative measurements of the nuclear cytoplasmic ratio of Pap1-GFP.

We agree that we have not tested exhaustively all known players involved in the cellular responses to (oxidative) stress, mostly due to practical reasons. Nevertheless, we have provided strong evidence against the activation of a general oxidative stress response:

- Sty1 and Atf1 are not hyperactivated in cbf11KO (Fig. 3A)
- Pap1 is not hyperactivated upon treatment with cerulenin, which does cause increased stress gene expression (Fig. 3C)
- using a very sensitive reporter (PMID: 31586057), we show that cbf11KO cells do not have increased ROS levels compared to WT (Fig. 1D), thus increased Pap1 activity is not expected in this mutant

As for any role of Cbf11 in regulating stress-responsive TFs:

- we only observed the mild upregulation of atf1 mRNA (~1.8x up) in cbf11KO cells, as mentioned in our manuscript
- mRNA levels of the pcr1, pap1, prr1 and hrr1 stress TFs are not upregulated in cbf11KO (PMID: 26366556)
- there is no Cbf11 binding to stress gene promoters, making it unlikely for Cbf11 to regulate stress TF recruitment (PMID: 26366556)
- as mentioned above, the interactions reported in the Pancaldi paper may be indirect (which is actually likely, given the broad range of apparent interaction strengths / MS peptide counts) (PMID: 22540037)
- most importantly, stress gene expression is upregulated also in Pcut6MUT or in cerulenin, where Cbf11 is intact (so the physical presence/absence of Cbf11 seems not to be important)

Also, does overexpression of cbf11+ cause hypersensitive growth and repression of oxidative stress gene expression in response to H2O2 treatment?

While we have not tested the sensitivity/resistance of cbf11OE cells to hydrogen peroxide, we note that mRNA levels of sty1, pap1, atf1, ctt1, srx1, pyp2, and gst2 are not downregulated upon cbf11 overexpression. But neither are there any changes in cut6 transcript levels in cbf11OE (PMID: 26366556). This is not completely unexpected, as we have shown previously that the phenotype of Cbf11 overexpression is not simply a mirror image of cbf11 deletion (PMID: 19101542).

Wild-type cells treated with cerulenin (inhibition of fatty acid synthesis independent of Cbf11) causes an induction of oxidative stress genes but is this regulation similar or distinct from Cbf11? Would wild-type cells exposed to cerulenin cause histone hyperacetylation at stress genes or is this response to compromised lipid biosynthesis only specific to Cbf11? Since cerulenin treatment appear to not involve Pap1 activation, are the other oxidative stress responsive transcription factors or Sty1 activated? Cerulenin treatment is transient and does not directly determine if compromised lipid biosynthesis independent of Cbf11 confers resistance to H2O2. It may be beneficial to determine if the fas1 deletion confers resistance to H2O2, as in S. cerevisiae and if oxidative stress response genes/regulators are induced in this genetic background.

These are valid and interesting questions and suggestions. But we strongly feel that getting the answers is beyond the scope of the current study. We believe that our current data form a coherent set that supports the conclusion that decreased FA synthesis leads to HAT-mediated resistance to ox. stress (without triggering a stress response as such). Therefore, we respectfully decline to perform the suggested additional experiments.

**Have all data underlying the figures and results presented in the manuscript been provided?**

Large-scale datasets should be made available via a public repository as described in the *PLOS Genetics* [data availability policy](http://journals.plos.org/plosgenetics/s/data-availability), and numerical data that underlies graphs or summary statistics should be provided in spreadsheet form as supporting information.

Reviewer #1: Yes

Reviewer #2: Yes

PLOS authors have the option to publish the peer review history of their article ([what does this mean?](https://journals.plos.org/plosgenetics/s/editorial-and-peer-review-process#loc-peer-review-history)). If published, this will include your full peer review and any attached files.

If you choose “no”, your identity will remain anonymous but your review may still be made public.

**Do you want your identity to be public for this peer review?** For information about this choice, including consent withdrawal, please see our [Privacy Policy](https://www.plos.org/privacy-policy).

Reviewer #1: **Yes:** Per Sunnerhagen

Reviewer #2: No
